# Development of a Machine Learning Model to Predict Recurrence of Oral Tongue Squamous Cell Carcinoma

**DOI:** 10.3390/cancers15102769

**Published:** 2023-05-16

**Authors:** Yasaman Fatapour, Arash Abiri, Edward C. Kuan, James P. Brody

**Affiliations:** 1 Department of Biomedical Engineering, University of California, Irvine, CA 92617, USA; yfatapou@uci.edu (Y.F.);; 2 Department of Otolaryngology-Head and Neck Surgery, University of California, Irvine, CA 92604, USA

**Keywords:** oral tongue squamous cell carcinoma, cancer recurrence, machine learning, oral cancer, artificial intelligence

## Abstract

**Simple Summary:**

In this study, we developed a generic framework to analyze the Surveillance, Epidemiology, and End Results (SEER) database to generate reliable machine learning (ML) prediction models for cancer recurrence. As a proof-of-concept, using 130,979 oral tongue squamous cell carcinoma patients, we generated ML models to predict 5- and 10-year recurrence with high accuracy, recall, and precision. Thus, we demonstrate an effective framework for guiding future ML efforts in predicting cancer recurrence using the SEER database, with implications for the guidance of patient management and follow-up care.

**Abstract:**

Despite diagnostic advancements, the development of reliable prognostic systems for assessing the risk of cancer recurrence still remains a challenge. In this study, we developed a novel framework to generate highly representative machine-learning prediction models for oral tongue squamous cell carcinoma (OTSCC) cancer recurrence. We identified cases of 5- and 10-year OTSCC recurrence from the SEER database. Four classification models were trained using the H_2_O ai platform, whose performances were assessed according to their accuracy, recall, precision, and the area under the curve (AUC) of their receiver operating characteristic (ROC) curves. By evaluating Shapley additive explanation contribution plots, feature importance was studied. Of the 130,979 patients studied, 36,042 (27.5%) were female, and the mean (SD) age was 58.2 (13.7) years. The Gradient Boosting Machine model performed the best, achieving 81.8% accuracy and 97.7% precision for 5-year prediction. Moreover, 10-year predictions demonstrated 80.0% accuracy and 94.0% precision. The number of prior tumors, patient age, the site of cancer recurrence, and tumor histology were the most significant predictors. The implementation of our novel SEER framework enabled the successful identification of patients with OTSCC recurrence, with which highly accurate and sensitive prediction models were generated. Thus, we demonstrate our framework’s potential for application in various cancers to build generalizable screening tools to predict tumor recurrence.

## 1. Introduction

Oral tongue squamous cell carcinoma (OTSCC) is a common head and neck neoplasm that accounts for approximately 1% of new cancer cases diagnosed in the United States each year [1]. Despite advancements in cancer therapeutics and surgical techniques, the worldwide incidence of OTSCC is on the rise, and adequate OTSCC management still remains a challenge, with 5-year survival rates for patients averaging at about 50% [2,3,4,5]. With recent studies reporting recurrence rates as high as 32.7%, further investigations aimed at optimizing treatment regimens and post-therapy follow-up are critical for enhancing patient outcomes [6,7,8]. 

The advent of machine learning (ML) and its adoption by the medical community has enabled unique perspectives and solutions for numerous medical challenges. Over the past decade, scientific efforts have demonstrated the utility of machine learning in guiding cancer diagnosis and management in a variety of medical fields, including general surgery, neurosurgery, and otolaryngology [9,10,11,12,13]. Specifically, many studies have applied machine learning techniques for predicting tumor diagnosis, tumor recurrence, and patient survival in the context of various cancers [14,15,16,17,18,19,20,21]. Recently, Alabi et al. and Karadaghy et al. demonstrated the capacity for ML to elucidate models and predict recurrence and survival, respectively, in OTSCC patients [22,23]. However, as with many of their predecessors, these studies are limited by the small samples of patients from which their models were trained.

Over the past two decades, a widespread shift toward the use of electronic medical records has resulted in a rapid accumulation of digital medical data, from which large administrative registries have been formed. The Surveillance, Epidemiology, and End Results (SEER) program, in particular, provides one of the largest cancer databases in the United States and represents nearly 48% of the national population. Recently, ML experts have been able to leverage the expansive nature of the SEER database to generate more precise and representative models to predict patient survival. However, to date, there is a paucity of studies that have attempted to utilize this database for predicting the recurrence of cancer following treatment and complete remission.

Thus, in this study, we developed a novel algorithm to identify cases of cancer recurrence in the SEER database, from which we generated ML models to accurately predict 5- and 10-year locoregional OTSCC recurrence. By using simple and commonly acquired prognostic markers as the basis of our models, we enabled our system to be more accessible and easily adoptable by a wide range of practitioners. Furthermore, we leveraged our nationally representative ML models to accurately classify patients into low- and high-risk categories. Hence, our system not only lays a foundation for future ML efforts in predicting cancer recurrence using the SEER database but also serves as an accurate data-driven tool for the prediction of OTSCC recurrence, with implications for the guidance of cancer management and follow-up medical care.

## 2. Methods

**Figure 1:**
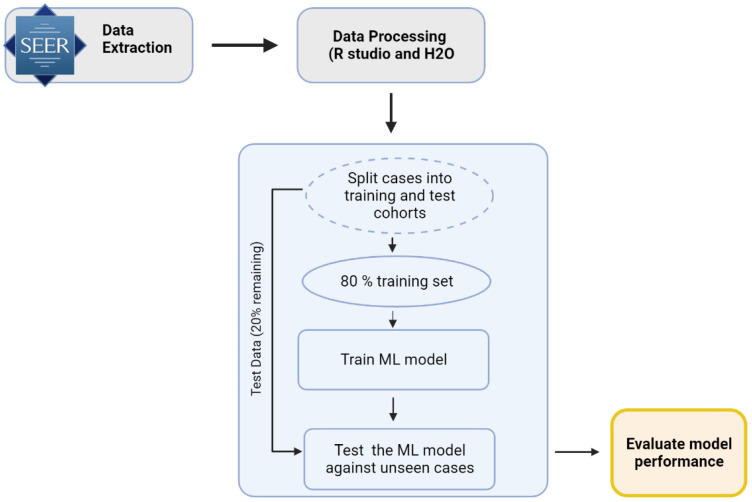
Schematic of data processing and model development. The model development process, data cleaning, and machine learning steps were performed in the R studio and H_2_O.ai tool.

A novel strategy was implemented for extracting cases from the SEER database with the goal of identifying the locoregional recurrence of cancer within 5-year and 10-year periods. As further detailed in the sections below, SEER*Stat version 8.3.9 (Surveillance Research Program, National Cancer Institute, Bethesda, MD, USA) was used to extract data from 18 SEER registries from 2000 to 2018. After processing the data and extracting variables of interest, the dataset grouping, feature extraction, and training and validation of the model were performed (Figure 1). 

### 2.1. Seer Database Query (Data Source) 

The 2000–2018 SEER database is a deidentified registry that reports cancer incidence and survival data on approximately 48% of the national population, serving as one of the largest and most comprehensive efforts for tracking oncological cases within the U.S. Due to the massive scale of available data, this work utilized SEER as its target database. Due to the anonymized and public nature of the SEER database, this study was exempt from the University of California Irvine Institutional Review Board’s approval.

The database was queried for patients diagnosed with OTSCC using the International Classification of Disease for Oncology, 3rd Edition (ICD-O-3) topography codes for the oral tongue (C02.0-C02.9) and histology/behavior codes for squamous cell carcinoma (SCC; 8010/3, 8020/3, 8021/3, 8070/3, 8071/3, 8072/3, 8073/3, 8074/3, 8082/3). The following demographic and clinical variables of interest were used to train our machine learning models for age, sex, race, marital status, year of diagnosis, the number of prior tumors, tumor site (e.g., ventral surface of the tongue, dorsal surface of the tongue, border of the tongue), histology, tumor grade, T/N/M stage, and administered treatments (i.e., surgery, radiation, chemotherapy). To account for variant-specific OTSCC behavior, histology was stratified into the following prognostic categories: nonkeratinizing SCC with maturation, undifferentiated nonkeratinizing SCC, differentiated nonkeratinizing SCC, and keratinizing SCC [24]. Furthermore, each case contained a sequence number that provided information on the number of all reportable primary tumors that occurred over the lifetime of a patient. This variable was used to calculate the “Number of prior tumors”, which was defined as the sequence number minus one. All cases with unknown or missing sociodemographic or outcome variables were excluded.

### 2.2. Patient Grouping and Feature Extraction 

Each individual case in the SEER dataset was defined by a unique patient identification number. The cases were first grouped according to their patient IDs before being subsequently sorted within their groups using their sequence numbers. Next, a series of validations were performed for all patients and their respective cases. These validations focused on minimizing errors in later classification steps by eliminating conditions where the “state of recurrence” (recurrence = true/false) could not be determined with absolute confidence based on the available SEER data. The following validations were implemented:

1. The oral tongue should be the primary site of the first case for each patient.
2. All cases corresponding to patients with missing or unknown values for any variable critical for analysis, including the Total Number of Malignant Tumors, Sequence Number, Survival Months, and Year of Diagnosis, were filtered out.

In the final step of the algorithm, we computed the target outcome variable, “Will Recure”. This variable, which was computed for each individual case, defined whether or not a case would recure in locoregional sites within the defined period of time (5 and 10 years). Of note, due to SEER coding guidelines, a recurrence that occurred at the exact same topographical code as its prior incident case was not reported in the database and, thus, was unavailable for analysis. A non-recurrence was defined as a patient that had only one primary tumor and survived longer than the target window (e.g., 5 years). Conversely, if there was another recurrence of cancer within the target window and in the same region as the initial tumor, then the case was marked “Will Recur” = true. It is worth noting that, based on the algorithm above, the last case for a patient with multiple primary tumors (i.e., multiple cases) would be marked as will not recur if the patient survived longer than the target window without another recurrence of cancer. This is critical as it tends to indicate successful treatment.

### 2.3. ML Training & Validation (Balancing, under Sampling, Number of Runs and Distribution of Data) 

We used the H_2_O AI platform (H_2_O.ai, Inc., Mountain View, CA, USA) in conjunction with an R statistical computing environment (version 3.6.1; The R Foundation for Statistical Computing) to train and test numerous machine learning models with the goal of identifying the best model for the prediction of the locoregional recurrence of OTSCC. In order to properly validate and test each model, the dataset was split into training (80%) and test (20%) sets. H_2_O’s Automl function was used to run through different machine learning algorithms and evaluate various hyperparameters for each algorithm [25]. Using the H_2_O Automl function, we trained and evaluated various machine learning algorithms, including the gradient boosting machine (GBM), distributed random forest (DRF), deep learning, logistics regression, and generalized linear model (GLM) [25,26,27,28,29,30,31]. To prevent overfitting during the training phase, we initially assessed the performance of these models using a 5-fold cross-validation technique. The trained models were ranked based on their AUC values, and we selected the top four models for further evaluation on an unseen data split.

The evaluation metrics, accuracy, precision, recall (sensitivity), and area under the curve (AUC) for the receiver operating characteristic (ROC) were computed for the top four predictive models on a separate 20% test set. The method to compute these hyperparameters is presented in Equations (1)–(3).

(1) $$Accuracy=\frac{\left(TP+TN\right)}{\left(TP+TN+FP+FN\right)}$$

(2) $$Precision=\frac{TP}{\left(TP+FP\right)}$$

(3) $$Recall=\frac{TP}{TP+FN}$$

Due to the unbalanced nature of the dataset, with fewer recurrence cases compared to non-recurrence cases (13,873 non-recurrence vs. 657 recurrence cases for 5 years and 6129 non-recurrences vs. 971 recurrence cases for 10 years), we evaluated two approaches for balancing the data. The first was oversampling, which involved synthesizing new examples from the existing samples for the minority class [22]. The downside of oversampling is that it introduces the risk of overfitting and/or introducing mathematically valid yet logically non-sensical sample sets. The second approach was under-sampling, which involved randomly selecting examples from the majority class to remove from the training dataset. In general, under-sampling is the preferred method, particularly for a large dataset [32,33]. In this case, the application of the massive SEER dataset helped make the utilization of the under-sampling approach a reality, further strengthening the accuracy of the final model. H_2_O was executed with a 5-fold cross-validation, which was configured for a maximum runtime of 600 s. For each ML model, 5 different runs were executed, and the average performances of the top four ML models were compared using the areas under the curves (AUCs) for the receiver operating characteristic (ROC) curves. 

## 3. Results

### 3.1. Study Population Characteristics and Cancer Recurrence Information

A total of 136,826 cases were extracted from the SEER dataset, which represented 130,979 unique patients. Two models were trained: one focusing on the locoregional recurrence of OTSCC in a 5-year period and the other in a 10-year period. In the 5-year analysis, 14,530 patients met the inclusion criteria, of which 657 suffered from a locoregional recurrence. For the 10-year analysis, 7100 patients met the inclusion criteria, of which 971 experienced a locoregional recurrence. It is worth noting that only patients alive within the follow-up period (5- or 10 years) were considered in our analyses. Table 1 shows a summary of predictors that were used to train the machine learning model.

### 3.2. Model Prediction and Development (Performance Metric for the Algorithm) 

**Figure 2:**
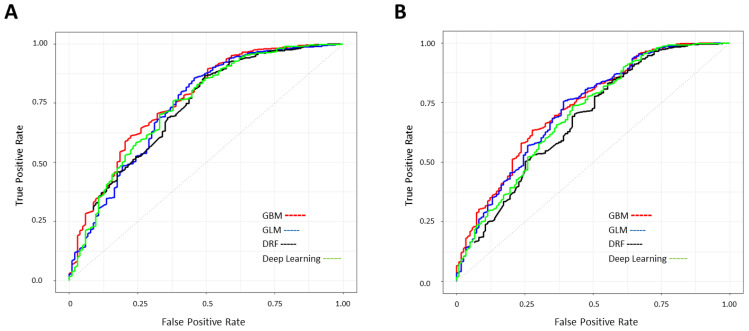
ROC plots of four developed ML models. Performance of Gradient Boosting Machine (GBM), Generalized Linear Model (GLM), Distributed Random Forest (DRF), and deep learning (artificial neural network) models in predicting (**A**) 5-year and (**B**) 10-year OTSCC recurrence. Patient’s data were split into an 80% training set and a 20% test set and 5-fold cross-validation was performed in each run.

To identify the most predictive model, the AUC of the ROC curve was used as a metric to compare the performance of four machine learning algorithms: Generalized Linear Model (GLM), Gradient Boosting Machine (GBM), Distributed Random Forest (DRF), and deep learning (artificial neural network, Figure 2) on the test split.

The performance metrics of the top four ML models are shown and compared in Table 2. The GBM classification model with an AUC of 0.75 (0.01) and 0.74 (0.02) outperformed all other models for both the 5-year prediction and 10-year prediction, respectively. Of note, the accuracy, recall, and precision of the model could be calculated at different thresholds within the graph of the ROC curve. Thus, the optimum threshold for each model varied depending on the definition and application of the classification problem. For example, a screening tool may require high recall and precision. For this proof-of-concept effort, we focused on using the model as a screening tool and, therefore, aimed to increase recall without a major sacrifice of accuracy. Therefore, the best overall performance for predicting OTSCC recurrence was achieved by the GBM model with 81.8% accuracy, 83.0% recall, and 97.7% precision for a 5-year prediction, and 80.0% accuracy, 82.8% recall, and 94.0% accuracy for 10-year prediction. 

**Figure 3:**
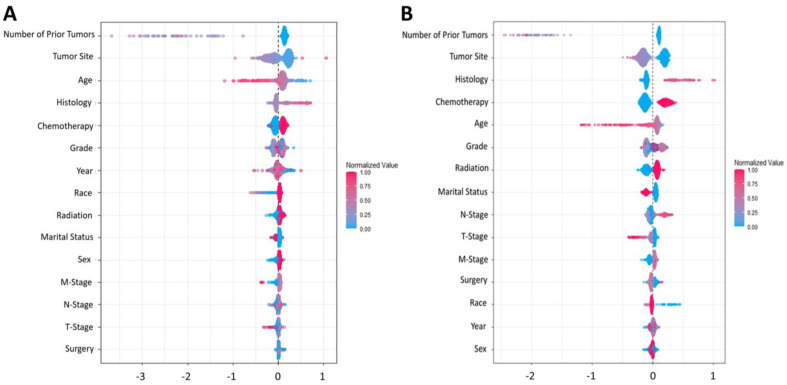
The Shapley Additive exPlanations contribution plots (SHAP) for the GBM model. SHAP plots of (**A**) 5-year and (**B**) 10-year prediction models. All pairs of coalitions between features of the ML model were calculated, and the feature’s importance was ranked from top to bottom.

In addition to the performance metrics of the model, we were also interested in the impact of each individual feature on the predictive outcome. The Shapley Additive exPlanations contribution plot (SHAP) illustrated how the GBM model arrived at its results (Figure 3) and explored the non-linearity effects on the features of this model [34]. It ranked (from top to bottom) the importance of each feature in a predictive model based on all the possible pairs of coalitions between predictors of the model. A higher importance score was indicative of a higher contribution to the model’s predictive ability. As shown, the number of prior tumors, age, and tumor site were the most important factors for determining the probability of the locoregional recurrence of OTSCC.

## 4. Discussion

In this study, we developed a novel framework with which to identify cases of cancer recurrence from the SEER database alongside generalizable and highly representative machine-learning models that could be generated. We demonstrated the utility of this framework by developing ML models that predicted 5- and 10-year cancer recurrence with high accuracy and precision using a large population-based cohort of OTSCC patients. Specifically, of the four ML algorithms that we employed, the GBM-based model showed the most promise, demonstrating accuracies of 82% and 80% for 5-year and 10-year recurrence, respectively. Of note, we observed a recurrence rate of ~5%, which was lower than the 16–33% recurrence rate that has been previously reported [8,35,36]. This was due to the stringent exclusion criteria that we applied, which required that patients with certain missing or unknown case information were excluded from the analysis. However, we do not anticipate this lower prevalence to have influenced our findings since, unlike traditional regression techniques that compute likelihood or risk scores based on a sample’s observed event rate, our machine learning model was trained using an under-sampling approach on the majority class (non-recurrence) in order to be tolerant of deviations from true population prevalence rates. Ultimately, by using simple and widely accessible demographic and clinical variables as the basis for model training, our sensitive prediction model showed promise in serving as a screening tool with which to assist clinicians in managing OTSCC patients during and after their treatment course.

Although significant progress has been made in cancer diagnostics and treatment, the prognosis of OTSCC is still poor, with many patients experiencing cancer recurrence and surviving less than 10 years after their initial diagnosis [23,37]. By developing a predictive screening tool, treatment teams can be better informed of a patient’s risk for cancer recurrence and modify their management strategy accordingly. Additionally, the mortality rate in recurrent cases of OTSCC is highly dependent on the time of diagnosis, with the early detection of recurrence being associated with reduced mortality [38,39]. By using our highly representative and sensitive classification models, clinicians can be better informed of which patients are at a higher risk of OTSCC recurrence and cater to their management and follow-up to ensure timely diagnosis if a recurrence were to occur.

In our analysis, we used SHAP to explain the predictions made by the Gradient Boosting model and interpret the tangled nonlinear relationships between the features and local regional recurrence of OTSCC. Consequently, we found that the number of prior tumors, patient age, tumor site, chemotherapy, tumor histology, and tumor grade were consistently the most influential features when predicting cancer recurrence. Thus, by developing an artificial intelligence (AI) model in the context of a highly representative population for cancer recurrence and analyzing the nonlinear effect of features through the SHAP method, we found some of the features to be more prognostic compared to those that were traditionally considered major prognostic factors in oral tongue cancer recurrence, such as lymphatic invasion or the T-stage [8,36]. Importantly, these findings do not discount the prognostic importance of previously reported clinical factors but rather highlight certain factors that may be generally considered highly prognostic across a more diverse and heterogeneous patient population.

In a recent institutional study, Alabi et al. similarly demonstrated success in predicting locoregional recurrence in OTSCC. However, despite their impressive results, their models were trained using only 217 cases of early-stage OTSCC, which largely limited their system’s applicability to more advanced tumors alongside its external validity against the general population, where the spectrum of disease behavior and progression is much more diverse than what is experienced at a single institution. Interestingly, the authors found that certain specialized histopathological parameters, such as lymphocyte host response, the pattern of invasion, depth of invasion, and perineural invasion, were particularly important features in their prediction models. Owing to the limitations of the SEER database, our models were trained without using these clinical features. While the lack of dependence on these specialized histopathological parameters expanded the accessibility of our system to a broader range of clinical facilities where such information may not be readily available, the consideration of these features may be warranted in future generations of ML models where a higher prediction accuracy in lieu of increased accessibility is desired.

Previous studies have reported on the significance of genetic predisposition in head and neck squamous cell carcinoma (HNSCC) [40,41]. Moreover, genetic and environmental factors, including a history of prior head and neck cancer, have been shown to be associated with the recurrence of HNSCC [42,43]. The influence of patient age on prognosis has also been previously established. In a large retrospective study of OTSCC patients, Mukdad et al. demonstrated that older patients were associated with more advanced disease and worse survival [5]. It was hypothesized that this worse prognosis was partly due to a tendency for clinicians to treat younger patients more aggressively with multimodality therapy. Interestingly, younger patients were also observed to less frequently present with metastatic lymph nodes. Indeed, survival and recurrence rates have been reported to be largely influenced by the presence of nodal disease [44]. As such, cancer recurrence at a regional site can be suggestive of a more aggressive disease with a tendency to recur following treatment. In a cohort study, Wolfer et al. suggested that aggressive neoplastic behavior is strongly dictated by tumor histology [45]. Specifically, the degree of keratinization in oral squamous cell carcinoma was demonstrated to be an important prognostic factor for recurrence and survival. Other recent studies have reached similar conclusions and have even created recurrence risk models on the sole basis of histological parameters [46,47,48,49].

To our knowledge, this is one of the first studies to develop an algorithm that can identify cases of cancer recurrence from the expansive and widely used SEER database, laying a basis for future investigations across a variety of medical fields. Through the use of this novel framework, we also present one of the first machine learning-based classification models that can accurately predict 5- and 10-year recurrence in OTSCC patients using only commonly available demographic and clinical features. 

There are, however, limitations to this study that are worth mentioning. Since patients were extracted from a de-identified national database, these data might be susceptible to information bias. Additionally, despite including a number of sociodemographic and clinical variables in our models, we would like to point out that certain potentially valuable histopathological (e.g., lymphocyte host response, perineural invasion, depth of invasion, tumor budding, and worst pattern of invasion) and clinical features (e.g., the timing of treatments, radiation dose, HPV status, neck dissection) were not accounted for due to the limitations of the SEER database. Despite these constraints, we were able to develop a model with high predictability for the locoregional recurrence of OTSCC. We believe that incorporating these site-specific variables along with other clinical and sociodemographic variables can only enhance the predictive power of these models [6,8,22,42,50]. We hope that this study will encourage the inclusion of such variables in future updates to SEER and other large-scale clinical datasets. Furthermore, we hope that this work will encourage future studies that focus on additional enhancements, such as hyperparameter tuning, increasing the training time, and utilizing alternative decision tree-based models such as xGBoost [51,52,53].

## 5. Conclusions

In this study, we developed a novel framework that could identify cases of cancer recurrence from the SEER database. Using a population-based sample of over 130,979 patients, we developed several highly accurate and sensitive machine-learning models to predict OTSCC recurrence. Despite the use of simple and commonly available prognostic markers as the sole features for our model training, the GBM-based model was nonetheless able to achieve prediction accuracies of 82% and 80% for 5- and 10-year cancer recurrence, respectively. With our framework’s ability to be applied to a wide variety of cancers, we believe that this tool can have significant implications in future oncologic research efforts that are aimed toward improving disease management and optimizing patient outcomes.

## Figures and Tables

**Table 1:** Summary of the sociodemographic and clinical predictors used in developing ML models for the prediction of OTSCC recurrence.

Variable	5-Year (N = 14,995)	10-Year (N = 7342)
	No. (%)	No. (%)
**Mean Age, years (SD)**	58.4 (11.5)	56.2 (11.5)
**Sex**		
Male	10,636 (72.0)	4075 (67.7)
Female	4129 (28.0)	1943 (32.3)
**Race**		
White	13,261 (89.8)	5991 (90.1)
Black	706 (4.8)	270 (4.1)
Asian	798 (5.4)	387 (5.8)
**Marital Status**		
Single	5056 (34.2)	2040 (30.7)
Married	9709 (65.8)	4608 (69.3)
**Number of Prior Tumors**		
0	14,051 (95.2)	6324 (95.1)
1	496 (3.4)	232 (3.5)
2	161 (1.1)	77 (1.2)
3	46 (0.3)	14 (0.2)
4+	11 (0.1)	1 (0.0)
**Histology**		
Nonkeratinizing SCC with maturation	11,468 (77.7)	5276 (79.4)
Undifferentiated nonkeratinizing SCC	86 (0.6)	39 (1.0)
Differentiated nonkeratinizing SCC	824 (5.6)	288 (4.3)
Keratinizing SCC	2286 (15.5)	993 (15.0)
SCC NOS	101 (0.7)	52 (1.0)
**Tumor Grade**		
Well-differentiated	2262 (18.8)	1067 (19.7)
Moderately differentiated	5752 (47.8)	2585 (47.6)
Poorly differentiated	3896 (32.4)	1710 (31.5)
Undifferentiated	117 (1.0)	64 (1.2)
**T-Stage**		
T1	4443 (46.7)	1594 (50.0)
T2	3274 (34.4)	1109 (34.8)
T3	1013 (10.6)	262 (8.2)
T4	784 (8.2)	221 (6.9)
**N-Stage**		
N0	5110 (45.5)	1918 (48.3)
N1	1968 (17.5)	764 (19.2)
N2	3847 (34.3)	1187 (29.9)
N3	296 (2.6)	102 (2.6)
**M-Stage**		
M0	11,200 (99.3)	3913 (99.2)
M1	75 (0.7)	30 (0.8)
**Surgery**		
Yes	6125 (41.8)	2506 (37.5)
No	8519 (58.2)	4185 (62.5)
**Radiation**		
Yes	8965 (60.7)	3811 (57.3)
No	5800 (39.3)	2837 (42.7)
**Chemotherapy**		
Yes	6598 (44.7)	2632 (39.6)
No	8167 (55.3)	4016 (60.4)

SCC: Squamous Cell Carcinoma; NOS: Not Otherwise Specified; values are based on the number of cases.

**Table 2:** Performance metrics of the top 4 machine learning models for predicting 5- and 10-year cancer recurrence. The GBM model exhibited the highest AUC and accuracy for both prediction windows.

Prediction Window	Classification Model	AUC (SD)	Accuracy % (95% CI)	Recall % (SD)	Precision % (SD)
5 Years	GBM	0.75 (0.01)	81.8 (79.7–83.9)	83.0 (0.02)	97.7 (0.002)
	GLM	0.73 (0.02)	77.4 (74.5–80.2)	78.1 (0.03)	98.0 (0.002)
	DRF	0.73 (0.03)	72.8 (69.8–75.7))	73.3 (0.02)	97.8 (0.003)
	Deep Learning	0.70 (0.04)	82.1 (74.7–89.6)	83.5 (0.06)	97.6 (0.002)
10 Years	GBM	0.74 (0.02)	80.0 [75.3, 84.1]	82.8 (0.04)	94.0 (0.004)
	GLM	0.73 (0.02)	78.4 [74.2, 82.7]	81.0 (0.04)	94.3 (0.002)
	Deep Learning	0.71 (0.02)	74.4 [70.1, 78.8]	76.6 (0.04)	94.0 (0.002)
	DRF	0.69 (0.01)	70.6 [68.0, 73.3]	72.2 (0.02)	93.8 (0.004)

AUC: Area Under Curve; GBM: Gradient Boosting Machine; GLM: Generalized Linear Model; DRF: Distributed Random Forest; performance metrics were reported as an average of five runs.

## Data Availability

The data that support the findings of this study are available from the corresponding author (JPB) upon reasonable request.
